# Reassessing the Role of the Type II MqsRA Toxin-Antitoxin System in Stress Response and Biofilm Formation: *mqsA* Is Transcriptionally Uncoupled from *mqsR*

**DOI:** 10.1128/mBio.02678-19

**Published:** 2019-12-17

**Authors:** Nathan Fraikin, Clothilde J. Rousseau, Nathalie Goeders, Laurence Van Melderen

**Affiliations:** a Cellular and Molecular Microbiology (CM2), Faculté des Sciences, Université Libre de Bruxelles (ULB), Gosselies, Belgium; University of Texas Southwestern Medical Center Dallas

**Keywords:** MqsR, MqsA, TA system, stress adaptation, biofilm, global regulation, persistence, RpoS, stress response, toxin-antitoxin

## Abstract

Toxin-antitoxin (TA) systems are broadly distributed modules whose biological roles remain mostly unknown. The *mqsRA* system is a noncanonical TA system in which the toxin and antitoxins genes are organized in operon but with the particularity that the toxin gene precedes that of the antitoxin. This system was shown to regulate global processes such as resistance to bile salts, motility, and biofilm formation. In addition, the MqsA antitoxin was shown to be a master regulator that represses the transcription of the *csgD*, *cspD*, and *rpoS* global regulator genes, thereby displaying a pleiotropic regulatory role. Here, we identified two promoters located in the toxin sequence driving the constitutive expression of *mqsA*, allowing thereby excess production of the MqsA antitoxin compared to the MqsR toxin. Our results show that both antitoxin-specific and operon promoters are not regulated by stresses such as amino acid starvation, oxidative shock, or bile salts. Moreover, we show that the MqsA antitoxin is not a global regulator as suggested, since the expression of *csgD*, *cspD* and *rpoS* is similar in wild-type and Δ*mqsRA* mutant strains. Moreover, these two strains behave similarly in terms of biofilm formation and sensitivity to oxidative stress or bile salts.

## Abstract (precis)

There is growing controversy regarding the role of chromosomal toxin-antitoxin systems in bacterial physiology. *mqsRA* is a peculiar toxin-antitoxin system, as the gene encoding the toxin precedes that of the antitoxin. This system was previously shown to play a role in stress response and biofilm formation. In this work, we identified two promoters specifically driving the constitutive expression of the antitoxin, thereby decoupling the expression of antitoxin from the toxin. We also showed that *mqsRA* contributes neither to the regulation of biofilm formation nor to the sensitivity to oxidative stress and bile salts. Finally, we were unable to confirm that the MqsA antitoxin is a global regulator. Altogether, our data are ruling out the involvement of the *mqsRA* system in Escherichia coli regulatory networks.

## INTRODUCTION

Toxin-antitoxin (TA) systems are small ubiquitous operons generally consisting of a toxic protein and an antitoxin that neutralizes the cognate toxin (for reviews, see references 1–3). Type II TA systems are characterized by a proteinaceous and labile antitoxin that neutralizes the toxin by direct interaction (4–6). Type II antitoxins often harbor intrinsically disordered domains, allowing their recognition and degradation by ATP-dependent proteases such as Lon or the Clp machinery (7–9). Antitoxins also generally carry a DNA binding domain that binds operators located in the promoter of the cognate TA operon and can thus act as autorepressors, alone or in complex with the toxin (10–12). Autoregulation is alleviated when an excess of toxin is present, allowing the production of compensatory antitoxin and homeostatic maintenance of a high antitoxin-to-toxin ratio (12–14).

TA systems are heavily represented in the accessory genome of most bacterial species, with representatives identified in plasmids, phages, transposons, superintegrons, and genomic islands (15, 16). They were first discovered on low-copy-number plasmids and were shown to participate in plasmid maintenance through a process known as postsegregational killing or addiction (17–20). However, most characterized and predicted TA systems are encoded in bacterial chromosomes and can constitute up to 4% of the total open reading frames (ORFs) in the genome of some species, as seen in Mycobacterium tuberculosis or Microcystis aeruginosa (21–23). The biological functions of these chromosomal TA systems remain ambiguous and are highly debated; previous studies reported a role for chromosomal TA systems in stress responses (24), programmed cell death (25), generation of specialized ribosomes (26), and antimicrobial persistence (27). However, contradictory studies failed to show an implication of TA systems in stress responses (28–30), programmed cell death (28, 31, 32), generation of specialized ribosomes (33, 34), or antimicrobial persistence (35–37). Other established functions in the literature, which are reminiscent of the mobile and addictive nature of TA systems, consist of stabilization of mobile genetic elements such as integrative conjugative elements (38) or superintegrons (39) but also protection against phages through abortive infection and against conjugative plasmids through anti-addiction and plasmid exclusion (40–42).

A well-characterized chromosomal TA system in Escherichia coli, *mqsRA*, has been associated with stress resistance (43, 44), biofilm formation (45, 46), and persister formation (47, 48). While most TA operons adopt a promoter-antitoxin-toxin organization, allowing the production of excess antitoxin supposedly through translational coupling, *mqsRA* adopts an unconventional promoter-toxin-antitoxin configuration (49–51). The MqsR toxin, an endoribonuclease, was shown to regulate motility (45), biofilm formation (45), and deoxycholate resistance (44). On the other hand, the MqsA antitoxin was described as a pleiotropic regulator that represses the expression of at least three known genes, as follows: *csgD*, which encodes a transcriptional regulator that modulates the production of cell surface-associated structures (i.e., curli fibers) and biofilm formation (46); *cspD*, which encodes a toxic protein linked with bacterial persistence (47); and *rpoS*, which encodes the general stress response sigma factor (43). MqsA was shown to be posttranslationally regulated by oxidative stress since hydrogen peroxide induces rapid degradation of the MqsA antitoxin by the Lon protease, leading to the derepression of MqsA-regulated genes like *rpoS* (43). Derepression of *rpoS* by MqsA would induce catalase expression and detoxification of reactive oxygen species, thus confirming the role of *mqsRA* as a stress response regulator (43). The autoregulatory properties of *mqsRA* differ from those of other TA systems since the MqsR toxin was shown to alleviate the binding of MqsA to the promoter of the system rather than act as a corepressor (52). Thus, a high level of production of MqsR could also result in the upregulation of MqsA-regulated genes (52). Transcript levels of *mqsR* were also shown to be upregulated by starvation in serine, valine, or glucose, although the mechanism of this upregulation is unknown (51).

Since the *mqsRA* system is organized in reverse order, i.e., with the toxin gene preceding the antitoxin one, it raises the question of how the expression of this system is regulated. We found that in addition to the promoter driving the expression of the *mqsRA* operon, *mqsA* transcription is driven by two constitutive promoters located in the *mqsR* coding sequence. These promoters are not regulated by MqsA and are together stronger than the promoter that drives the expression of the whole operon, suggesting that the expression level of the antitoxin exceeds that of the toxin. While investigating the signals that might regulate the transcription activity of the three promoters, we failed to demonstrate any regulation of these promoters by previously reported stress conditions. We also could not show any effect of *mqsRA* and other TA systems on resistance or tolerance to oxidative stress and deoxycholate or on biofilm formation.

## RESULTS

### Newly identified promoters uncouple *mqsA* transcription from *mqsR*.

**FIG 1:**
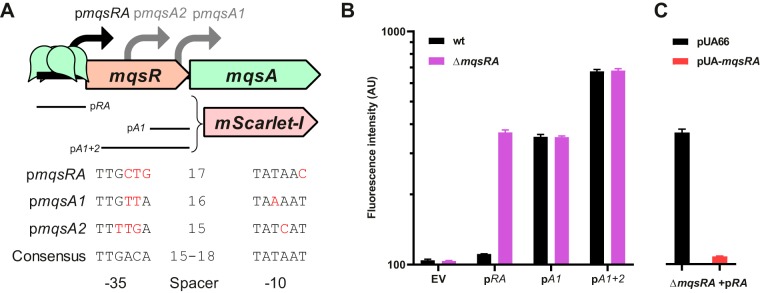
Transcription of *mqsA* is driven by two promoters located in the *mqsR* ORF. (A) Structure of the *mqsRA* operon and its promoters. Fragments used to construct transcriptional fusions are represented. The sequence of each promoter associated with an identified TSS is represented with divergences from the consensus in red. (B and C) Activity of *mqsRA* transcriptional fusions in the wild-type and Δ*mqsRA* mutant strains (B) or when complementing the *mqsRA* deletion (C). Cells grown to exponential phase (OD_600_, 0.4) were analyzed by flow cytometry. Data represent the geometric means of the results from three independent experiments in which the geometric mean fluorescence of 10,000 events was measured. Bars show standard deviations. EV, empty vector.

Due to the reverse configuration of the *mqsRA* system, we reasoned that additional promoters might be present in the *mqsRA* operon to allow excess antitoxin production. Putative promoters were predicted using the bprom software (53). A potential sigma70-binding site located inside the *mqsR* ORF (p*A1*) that could drive the expression of *mqsA* independently of *mqsR* was identified (Fig. 1A; see also Fig. S1A in the supplemental material). RNA sequencing data sets characterizing transcription start sites (TSS) in E. coli corroborate the functionality of this promoter, as a TSS can be detected 8 nucleotides (nt) downstream of this putative promoter (54). Another TSS can be found 111 nt upstream of this first TSS (54). A fairly conserved sigma70-binding sequence (p*A2*) can also be found 8 nt upstream of this second TSS (Fig. 1A and S1A).

10.1128/mBio.02678-19.1 FIG S1 Transcription of *mqsA* by two newly identified promoters. (A) Sequence of the *mqsRA* system in E. coli MG1655 (GenBank accession no. U00096.3). Promoter elements are represented inside a frame. Coding and protein sequences for *mqsR* and *mqsA* are represented in red and green, respectively. Palindromic operators binding MqsA are represented in blue. Putative ribosome binding sites are underlined. Fragments used to construct transcriptional fusions are shown with dashed lines. (B) Fluorescence distribution of a representative replicate from Fig. 1B and C. Download FIG S1, EPS file, 0.7 MB. Copyright © 2019 Fraikin et al. 2019 Fraikin et al. This content is distributed under the terms of the Creative Commons Attribution 4.0 International license.

To test whether these putative promoters are transcriptionally active, two fragments containing either p*A1* or p*A2* plus p*A1* (referred to as p*A1 + 2*) and the native *mqsA* ribosome binding site were cloned in a single-copy reporter plasmid, upstream of *mScarlet-I*, which encodes a fast-maturing and bright-red fluorescent protein (55) (Fig. 1A and S1A). These constructs were introduced in the MG1655 strain and the isogenic Δ*mqsRA* mutant. Fluorescence was analyzed by flow cytometry to study the effect of autoregulation on these two promoters as well as on the promoter driving the transcription of the full-length messenger that encodes the whole system (referred to as p*RA*).

Our results show that the p*RA* promoter has very low transcriptional activity, as this construct shows fluorescence (111 ± 1 arbitrary units [AU]) very close to the background level (104 ± 1 AU) (Fig. 1B). The activity of the p*RA* promoter is higher in the Δ*mqsRA* mutant strain (369 ± 9 AU), confirming that MqsA acts as a transcriptional repressor of this promoter (Fig. 1B). The repression of p*RA* in the Δ*mqsRA* mutant strain is restored by introducing the *mqsRA* operon in *trans*, confirming that this system is autoregulated (Fig. 1C). The p*A1* and p*A1 + 2* fragments showed stronger activities than did p*RA*, with p*A1* rivaling a derepressed p*RA* (354 ± 8 AU) and p*A1 + 2* being twice as strong (675 ± 12 AU), confirming the functionality of these predicted promoters in the *mqsR* ORF (Fig. 1B). However, deleting *mqsRA* had no effect on the transcriptional activity of p*A1* (352 ± 5 AU) or p*A1 + 2* (680 ± 13 AU), showing that these promoters are not subjected to autoregulation (Fig. 1B). Note that in all of these cases, the fluorescence distribution of the population was monodisperse (Fig. S1B).

### Stress does not regulate *mqsRA* at the transcriptional and posttranslational levels.

Since *mqsA* transcription appears to be mainly driven by two internal promoters under the testing conditions described above, we investigated whether these promoters could be regulated by different signals and therefore lead to the modulation of the ratio of toxin to antitoxin proteins. The transcriptional activities of p*RA*, p*A1*, and p*A1 + 2* were measured under various stress conditions previously assayed in the literature, i.e., amino acid starvation (51) and oxidative stress (43). Cells were starved for serine and isoleucine by using serine hydroxamate or valine, respectively, while oxidative stress was investigated using hydrogen peroxide and methyl viologen. Hydrogen peroxide induced rapid bleaching of the mScarlet-I reporter and is not presented here. Deoxycholate, a bile salt whose resistance is mediated by MqsR (44), was also investigated for its effects on these promoters.

**FIG 2:**
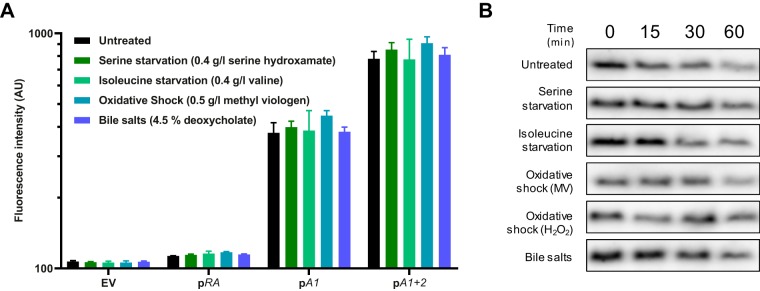
*mqsRA* is not regulated by stress. (A) Activity of *mqsRA* transcriptional fusions under stress conditions in wild-type E. coli MG1655. Cells grown to exponential phase (OD_600_, 0.4) were treated with 0.4 g/liter serine hydroxamate (serine starvation), 0.4 g/liter valine (isoleucine starvation), 0.5 g/liter methyl viologen (oxidative shock), or 4.5% sodium deoxycholate (bile salts) or left untreated for 60 min and analyzed by flow cytometry. Data represent the geometric means of the results from three independent experiments in which the geometric mean fluorescence of 10,000 events was measured. Bars show standard deviations. Two-way analysis of variance did not detect significant differences between untreated and treated conditions (*F* = 1.37, *P* = 0.36). EV, empty vector. (B) Degradation of MqsA under stress conditions. Wild-type MG1655 cells overexpressing *mqsA* grown to exponential phase (OD_600_, 0.4; with 0.5 mM IPTG) were treated with rifampin (200 μg/ml) and the indicated stress at time zero, sampled at indicated time points, and analyzed by Western blotting with an anti-MqsA antibody. MV, methyl viologen (0.5 mg/ml); H_2_O_2_, hydrogen peroxide (20 mM).

Surprisingly, none of the studied promoters, including p*RA*, showed appreciable changes in activity after 1 h of treatment with serine hydroxamate, valine, methyl viologen, or deoxycholate compared to activity under untreated conditions (*P* = 0.36) (Fig. 2A). Fluorescence distribution did not change under the treated conditions either (Fig. S2A). Upregulation of p*RA* under serine starvation was also not observed under conditions where *mqsR* was previously shown to be upregulated, i.e., M9 medium with glucose and amino acids (51) (Fig. S2B).

10.1128/mBio.02678-19.2 FIG S2 *mqsRA* is not regulated by stress. (A) Fluorescence distribution of a representative replicate from Fig. 2A. The concentrations are 0.4 g/liter serine hydroxamate (SHX), 0.4 g/liter valine (Val), 0.5 g/liter methyl viologen (MV), and 4.5% sodium deoxycholate (DoC). (B) Activity of the p*RA* reporter under conditions described by Christensen-Dalsgaard et al. (51). Wild-type cells carrying the p*RA* reporter were grown to exponential phase (OD_600_, 0.2) in M9 medium supplemented with 0.2% glucose and 0.04 g/liter each proteinogenic amino acid except l-serine. Serine hydroxamate (0.4 g/liter) was then added for 1 h before measuring fluorescence by flow cytometry. Bar shows standard deviation over three independent replicates. (C) Total protein Ponceau staining (top) and original Western blot (bottom) used to generate Fig. 2B. Arrow indicates the position of MqsA, which corresponds to its expected molecular weight of 15 kDa. SHX, serine hydroxamate, MV, methyl viologen. (D) Western blot showing MqsA stability under conditions described by Wang et al. (43). Cells were grown in LB medium to an OD_600_ of 0.1, and then 0.5 mM IPTG was added. At an OD_600_ of 1.0, rifampicin (200 μg/ml) and hydrogen peroxide (20 mM) were added. Protein extraction and subsequent steps were performed as in Fig. 2B. Download FIG S2, EPS file, 5.9 MB. Copyright © 2019 Fraikin et al. 2019 Fraikin et al. This content is distributed under the terms of the Creative Commons Attribution 4.0 International license.

MqsA stability was also assessed during previously mentioned treatments. Since our anti-MqsA antibody did not detect endogenous MqsA, a low-copy-number plasmid bearing the *mqsA* gene under the inducible *lac*L8.UV5 promoter was used to mildly overproduce MqsA. Our results show that MqsA is moderately unstable but is not degraded at a different rate when cells are treated with serine hydroxamate, valine, methyl viologen, hydrogen peroxide, or deoxycholate (Fig. 2B and S2C). Using conditions identical to those previously published, i.e., growth in LB medium (43), we also failed to observe degradation of MqsA in response to hydrogen peroxide (Fig. S2D). These unexpected results question the stress-responsive nature of *mqsRA* that was previously described in the literature (43).

### The MqsA antitoxin is not a pleiotropic regulator.

**FIG 3:**
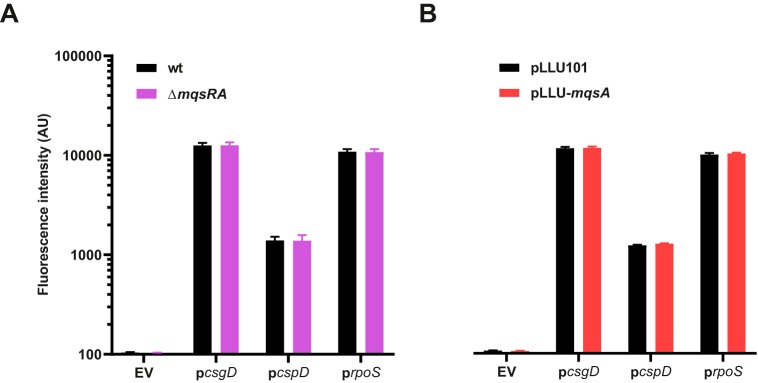
*mqsRA* does not regulate expression of the *csgD*, *cspD*, and *rpoS* genes. (A and B) Activity of *csgD*, *cspD*, and *rpoS* transcriptional fusions in a Δ*mqsRA* mutant (A) or under *mqsA* overexpression conditions (B). Cells grown to exponential phase (OD_600_, 0.4; with 0.5 mM IPTG in panel B) were analyzed by flow cytometry. Data represent the geometric means of the results from three independent experiments in which the geometric mean fluorescence of 10,000 events was measured. Bars show standard deviations. wt, wild type. EV, empty vector.

MqsA was shown to bind and repress several promoters, including *csgD*, *cspD*, and *rpoS* promoters, allowing this antitoxin to regulate biofilm formation, persistence, and the stress response (43, 46, 47). Since these regulations were demonstrated by overproducing ample amounts of the MqsA antitoxin (43, 46, 47), which can potentially lead to unspecific binding, repression of these promoters was investigated under more relevant conditions, especially since *mqsA*-binding operators found in these promoters significantly differ from those found in p*RA* (Fig. S3A). Transcriptional reporters of *csgD*, *cspD*, and *rpoS* promoter activities were constructed and validated by measuring their activities under conditions known to affect the transcription of these genes, as follows: p*csgD* is downregulated in high-osmolarity medium (56), p*cspD* is upregulated upon glucose starvation (57), and p*rpoS* is upregulated in an Δ*arcA* mutant during growth in LB medium (58) (Fig. S3B). Our results show that the activities of these three constructs are similar in wild-type and Δ*mqsRA* mutant strains (Fig. 3A). Mild overproduction of MqsA under a *lac*L8.UV5 promoter was used to assess whether an excess of MqsA would repress these three promoters. However, promoter activities showed no appreciable changes when MqsA was overproduced (Fig. 3B). No changes in fluorescence distribution were observed under these conditions (Fig. S3C). Altogether, these results show that MqsA does not regulate the activity of the *csgD*, *rpoS*, and *cspD* promoters as previously shown (43, 46, 47).

10.1128/mBio.02678-19.3 FIG S3 *mqsRA* does not regulate the expression of *csgD*, *cspD*, and *rpoS.* (A) Operators bound by MqsA described in the literature. Divergences from O1*_mqsRA_* are represented in red. (B) Validation of p*csgD*, p*cspD*, and p*rpoS* fluorescent reporters. p*csgD* was tested by growing wild-type cells bearing the reporter in high-osmolarity medium (MOPS-glucose with or without 8% sucrose) as in reference 9. p*cspD* was tested by growing wild-type cells containing the reporter in MOPS medium containing 0.4% glucose or 0.02% glucose (glucose starvation) as in reference 10. p*rpoS* was tested by growing wild-type or Δ*arcA* mutant cells containing the reporter in LB medium to exponential phase (OD_600_, 0.4) as in reference 11. Data represent the geometric means of the results from three independent experiments in which the geometric mean fluorescence of 10,000 events was measured. Bars show standard deviations. (B) Fluorescence distribution of a representative replicate from Fig. 3A and B. Download FIG S3, EPS file, 0.5 MB. Copyright © 2019 Fraikin et al. 2019 Fraikin et al. This content is distributed under the terms of the Creative Commons Attribution 4.0 International license.

### *mqsRA* and other TA systems do not foster stress resistance or tolerance.

**FIG 4:**
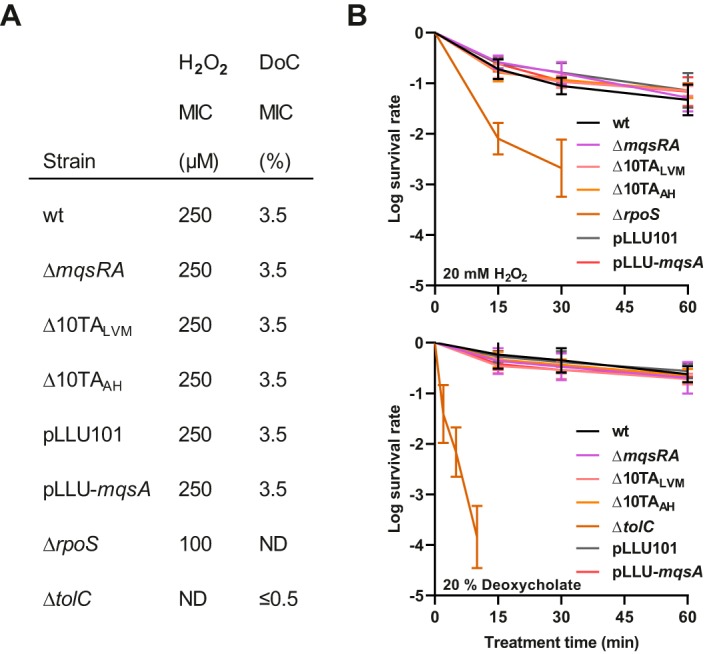
*mqsRA* does not play a role in oxidative stress and bile salts tolerance or resistance. (A) MICs of hydrogen peroxide and sodium deoxycholate of *mqsRA* and TA deletion mutants as well as under *mqsA* overexpression conditions. Exponentially growing cultures were diluted and spotted on M9 gluconate medium (with 0.5 mM IPTG for pLLU101 and pLLU-*mqsA*) with linear increases in peroxide or deoxycholate concentration. MICs were determined as the minimal concentration inhibiting growth. DoC, sodium deoxycholate; ND, not determined. (B) Killing kinetics of hydrogen peroxide and sodium deoxycholate of *mqsRA*, TA deletion mutants, and under *mqsA* overexpression conditions. Cells grown to exponential phase (OD_600_, 0.4; with 0.5 mM IPTG for pLLU101 and pLLU-*mqsA*) were treated with 20 mM hydrogen peroxide or 20% sodium deoxycholate at time zero. Samples at given time points were diluted and plated to determine the survival rate. Data represent the geometric means of the results from three independent experiments. Bars show standard deviations.

Since *mqsRA* was shown to play a pivotal role in survival to oxidative stress (43) and bile salts treatments (44), resistance and tolerance to hydrogen peroxide and sodium deoxycholate of a Δ*mqsRA* mutant strain were measured and compared to the wild-type strain. These experiments were also performed with two independently constructed Δ10TA mutant strains deleted for 10 type II TA systems, *mqsRA* included (35, 36). The minimal inhibitory concentrations (MICs) of peroxide and deoxycholate are similar in the wild-type, Δ*mqsRA* mutant, and Δ10TA mutant strains (Fig. 4A). Overexpression of *mqsA* did not affect peroxide or deoxycholate sensitivity either (Fig. 4A). Furthermore, no appreciable differences in survival were observed in a Δ*mqsRA* mutant or in Δ10TA mutants compared to the wild-type strain (Fig. 4B). Overexpression of *mqsA* did not affect the killing rate by these two compounds either (Fig. 4B). We used Δ*rpoS* and Δ*tolC* mutants as internal controls since they are hypersensitive to peroxide and deoxycholate, respectively (59, 60). Catalase activity, which was shown to be regulated by MqsA through RpoS (43), was also measured in exponentially growing cultures, and no change in activity was detected when *mqsRA* and other TA systems were deleted or when *mqsA* was overexpressed (Fig. S4). Thus, our results show that *mqsRA* and other TA systems do not play a role in tolerance or resistance to oxidative stress and bile salts as previously shown (43, 44).

10.1128/mBio.02678-19.4 FIG S4 *mqsRA* and other TA systems do not impact catalase activity. Cells were grown to exponential phase and treated with 60 mM hydrogen peroxide before sampling cultures at various time points and titrating unconsumed hydrogen peroxide with acidic dichromate. Data represent the means of the results from three independent experiments. Bars show standard deviations. Download FIG S4, EPS file, 0.3 MB. Copyright © 2019 Fraikin et al. 2019 Fraikin et al. This content is distributed under the terms of the Creative Commons Attribution 4.0 International license.

### *mqsRA* and other TA systems do not regulate biofilm formation.

**FIG 5:**
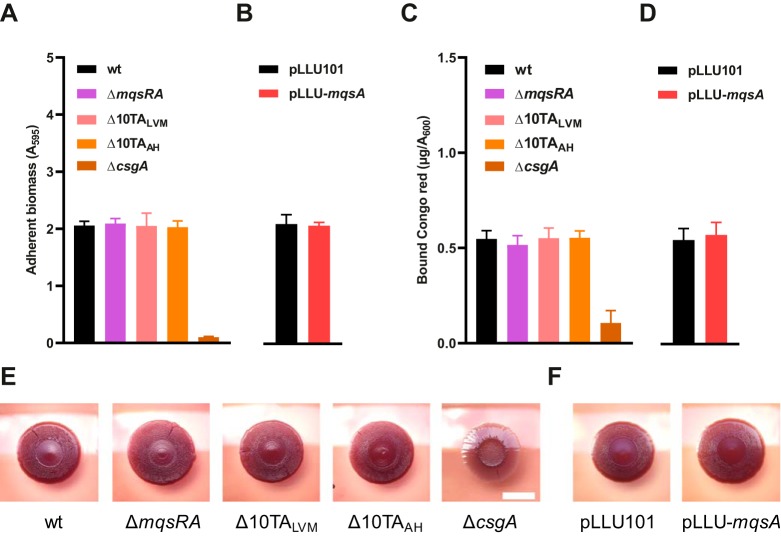
*mqsRA* and other TA systems do not play a role in biofilm formation. (A and B) Microplate adhesion assays of *mqsRA* and TA deletion strains (A) and under *mqsA* overexpression conditions (B). Cells were grown statically in 96-well plates (with 0.5 mM IPTG in panel B), and nonadherent cells were washed before staining adherent cells with crystal violet and measuring the absorbance at 595 nm (*A*_595_). Data represent the means of the results from three independent experiments. The results from each independent experiment are expressed as the mean from six technical replicates. Bars show standard deviations for the mean values from the three independent experiments. (C and D) Congo red binding of planktonic cells in *mqsRA* and TA deletion strains (C) and under *mqsA* overexpression conditions (D). Overnight cultures were washed, resuspended in 1% tryptone with 40 μg/ml Congo red (with 0.5 mM IPTG in panel D), and grown for 3 h. Free Congo red was measured in culture supernatants by spectrophotometry to determine the fraction of Congo red bound to cells, normalized to the turbidity of the culture. Data represent the means of the results from three independent experiments. Bars show standard deviations. (E and F) Macrocolonies of *mqsRA* and TA deletion mutants (E) or under *mqsA* overexpression conditions (F). Overnight cultures were spotted on Congo red agar (with 0.5 mM IPTG in panel F) and grown for 7 days at 28°C. Scale bar = 1 cm.

The role of *mqsRA* and other TA systems in biofilm formation was also reassessed since *mqsRA* was shown to regulate adherence (45) and the production of curli, amyloid fibers that play a pivotal role in biofilm formation and multicellular behaviors (46). Our results show that the Δ*mqsRA* and Δ10TA mutant strains generate similar amounts of adherent biomass as the isogenic wild-type strain (Fig. 5A). Moreover, mild overexpression of *mqsA* did not affect the amount of adherent biomass either (Fig. 5B). The presence of curli was quantified by measuring the binding of Congo red to planktonic cells. No differences in Congo red binding were found between the wild-type, Δ*mqsRA* mutant, and Δ10TA mutant strains (Fig. 5C) or upon mild overexpression of *mqsA* (Fig. 5D). In a macrocolony biofilm model, which consists of week-old colonies grown on a low-osmolarity medium containing Congo red (61), no morphological differences between the wild-type, Δ*mqsRA* mutant, and Δ10TA mutant strains, as well as no differences in coloration, were observed (Fig. 5E). Overexpression of *mqsA* did not affect these parameters either (Fig. 5F). A curli-deficient mutant (Δ*csgA*) was used as a control in all these experiments since it poorly forms biofilms and does not bind Congo red. Altogether, these results suggest that *mqsRA* and other type II toxin-antitoxin systems do not play a role in biofilm formation as previously shown (45, 46).

## DISCUSSION

Toxin-antitoxin systems have been attributed various biological functions ranging from antibiotic persistence to the generation of specialized ribosomes (24–27). However, in the last few years, numerous publications have questioned these findings and started substantial controversy over the role of TA systems in bacterial physiology (29–37).

In this work, we provide evidence that the *mqsRA* type II toxin-antitoxin system does not play a role in the stress response and biofilm formation as previously shown (43–47). Our results show that under all of the stress conditions in which *mqsRA* has been suggested to play a role, the transcription of both *mqsR* and *mqsA* remains stable. The half-life of the MqsA protein also remains unchanged under these stress conditions. It was hypothesized that MqsA regulates stress responses by repressing the transcription of the *rpoS* general stress sigma factor gene (43). Our results show that MqsA levels have no effect on *rpoS* expression. We also showed that deleting *mqsRA* has no effect on resistance or tolerance to two of these stresses which are bactericidal, namely, oxidative stress and bile salts. Therefore, we conclude that the *mqsRA* system does not respond to these various stress conditions and is likely not implicated in a response to these stresses. As no changes in *mqsA* expression under stress conditions were observed, both at the transcriptional and posttranslational levels, it is likely that MqsA is maintained at a steady and constant level. This is corroborated by the constitutive nature of the two newly identified *mqsA* promoters which would allow for a constant and steady level of *mqsA* transcription.

We also showed that *mqsRA* and nine other type II TA systems (*relBE*, *yefM-yoeB*, *mazEF*, *dinJ-yafQ*, *yafNO*, *chpB*, *higBA*, *hicAB*, and *prlF-yhaV*) do not play a role in biofilm formation using two different biofilm models, i.e., adherence in microplates and macrocolonies, and by showing that *mqsA* does not regulate *rpoS* and *csgD* expression, which are two pivotal factors in biofilm physiology (62). Moreover, we showed that *mqsRA* does not affect the biosynthesis of curli fibers, which are a pivotal component of the extracellular matrix in biofilms (61, 62).

In light of these contradictory results, we reexamined the literature covering the biological functions of *mqsRA*. The *mqsR* (motility and quorum-sensing regulator) gene was first identified as an autoinducer 2-responsive regulator of motility and biofilm formation (45). Indeed, it was shown that a Tn*5* insertion mutant of *mqsR* was less motile and formed less adherent biomass (45). Interestingly, this low-motility phenotype could be complemented by overexpressing *mqsR* under a strong inducible promoter on a multicopy plasmid compared to a wild-type strain (45), despite being later shown to be a growth-inhibiting toxin (50). It was also reported that the effects of *mqsR* on motility and biofilm formation were mediated through the *qseBC* two-component system, which is located around 1 kb upstream of *mqsR* (45). We hypothesize that a Tn*5* insertion in *mqsR* could alter *mqsRA*-mediated transcriptional regulation of the p*RA* promoter and induce polar effects in its close vicinity. The presence of an *frt*-flanked kanamycin resistance cassette or an *frt* scar *in lieu* of *mqsR* had contradictory effects on biofilm formation, supporting the hypothesis that a polar effect might be responsible for these effects on motility and biofilm formation (50). It was also reported that deleting *mqsRA* in a strain already deleted for five TA systems increased biofilm formation instead of reducing it (43). Another explanation for these discrepancies would be that the aforementioned MG1655 *mqsR*::Tn*5* strain is not isogenic to the wild-type strain to which it was compared in this study (45). Indeed, this *mqsR* mutant shows a drastic decrease in the levels of Crl (26-fold), an RNA polymerase-RpoS holoenzyme assembly factor responsible for curli biosynthesis and biofilm formation. It is known that various clones that are labeled as MG1655 show genetical differences, especially with regard to insertion sequence (IS) dynamics (63). The *crl* gene is known to be disrupted by an IS*1* insertion in a specific clone of MG1655, CGSC7740 (64, 65). Increased expression of the *opp* and *glp* operons as well as reduced *gatC* expression were also observed in the *mqsR*::Tn*5* mutant when compared to a wild-type strain (45). The CGSC7740 clone harbors several mutations that can explain these differences. First, an IS*5* insertion in the *opp* promoter disrupts an operator known to be bound by the Lrp repressor, thus likely causing increased expression of this operon (65). A nonsense mutation in the *glpR* gene encoding the repressor of the *glp* operon can also be found in this clone and is known to induce derepression of the *glp* operon (65, 66). Finally, another nonsense mutation can be found in the *gatC* gene, thus likely causing reduced expression of this gene (65). Also, many transcripts from the *e14* cryptic prophage (*ymfI*, *lit*, *ymfD*, *ymfK*, *ymfG*, *ymfJ*, *ymfT*, *ymfL*, *intE*, and *mcrA*) are detected more abundantly in the *mqsR*::Tn*5* mutant (45). Since *e14* can readily excise from its attachment site (67), this could suggest that this prophage was excised from the wild-type strain that was compared to the *mqsR*::Tn*5* mutant. An apparent decrease in *flhDC* expression in the *mqsR*::Tn*5* mutant, and thus, reduced motility, can also be explained by the high propensity of the *flhDC* promoter to undergo IS*1*/IS*5* insertions and excisions, which are known to affect motility in supposedly isogenic strains (63, 64, 68). Altogether, we suggest that the *mqsR*::Tn*5* mutant might have been constructed from clone CGSC7740 but compared to another, nonisogenic wild-type MG1655 strain. Differences that were observed between these two strains are likely due to differences in background rather than to the disruption of *mqsR* since motility and biofilm formation are known to vary between various backgrounds of E. coli K-12 (45, 50, 64, 69). This is also supported by the fact that we did not observe changes in any protein levels in strains deleted for 10 TA systems, including *mqsRA*, compared to an isogenic wild type (36). Other sources for discrepancies could be phase variations; indeed, *mqsR* was previously shown to regulate the *flu* gene encoding the antigen 43 adhesin (45, 47). However, a subsequent study showed that *mqsR* does not regulate *flu* and that phase variations in activation of the *flu* promoter through *dam* methylation could be responsible for these contradictory reports (70).

Another study showed that *mqsA* regulates biofilm formation by repressing the transcription of the *csgD* master regulator of curli biosynthesis (46). This was shown by overexpressing *mqsA* using high-copy-number vectors, such as pBS(Kan) *mqsA* (46), a Bluescript-based vector with a *lac* promoter (71), or pCA24N *mqsA* (43, 47), a *rop*-pBR322-based vector with a T5-*lac* promoter (72). These vectors are replicated by origins that are mutant derivatives of the pMB1 replicon, which are known to be maintained at several hundreds of copies per cell (71, 72). Using such high-copy-number vectors could lead to a disproportionate production of MqsA, which would bind to low-affinity and biologically irrelevant operator-like sequences like the ones present in the promoters of *csgD*, *cspD*, and *rpoS*. Using a pSC101-based vector (5 copies per chromosome) as well as a deletion mutant of *mqsRA*, we showed that MqsA levels, whether they are reduced by deletion or increased by reasonable overexpression, do not affect the transcriptional activities of the *csgD*, *cspD*, and *rpoS* promoters.

A gene found directly downstream of *mqsRA*, *ygiS*, was shown to be an important factor for deoxycholate resistance (44). The *ygiS* transcript, encoding a protein promoting deoxycholate import, is cleaved by MqsR, thus promoting its downregulation and reducing deoxycholate import and sensitivity (44). However, we failed to show that *mqsRA* was responsive to deoxycholate at both the transcriptional and posttranslational levels or had any effect on deoxycholate tolerance or resistance. Since *ygiS* is located directly downstream of *mqsRA*, we cannot exclude the possibility that the *mqsRA* deletion performed by the authors had polar or readthrough effects on *ygiS* transcription. It is also worth to note that the overexpression of *mqsR* induces cleavage of 30% of the messenger transcriptome as well as in rRNA precursors, showing that *mqsR* is an RNase promoting general downregulation of translation by cleaving RNA indiscriminately rather than being a posttranscriptional regulator that cleaves specific transcripts to promote their downregulation (34, 73). Moreover, the overexpression of *mqsR* was shown to increase *ygiS* transcript abundance rather than decrease it (34).

Another publication showed that *mqsA* was mediating the general stress response by repressing *rpoS* transcription (43). Under oxidative stress, MqsA was degraded by the Lon protease, leading to derepression of *rpoS* and increased stress resistance (43). However, this regulation was tested again by using ample overexpression of 6×His-MqsA using a pCA24N vector. As stated above, overproduction studies are not an appropriate experimental setup. Moreover, the authors showed that 6×His-MqsA overexpressed using this vector was rapidly degraded under oxidative stress, contrary to what we showed with reasonably overexpressed and untagged MqsA. The reason of this discrepancy is unclear, but the presence of a His tag or the high level of production, which might possibly induce misfolding, could increase the proteolytic sensitivity of MqsA under oxidative stress.

The idea that toxin-antitoxin systems respond to stress and regulate biological processes is not recent (24, 25, 74). For example, many TA systems in E. coli have been shown to be transcriptionally upregulated under stress conditions (e.g., serine hydroxamate-induced amino acid starvation) (51, 75). This has been attributed to stress-induced degradation of antitoxins, leading to the derepression and transcriptional upregulation of TA operons. This would allow the liberation of toxins from TA complexes, which would then be able to exert bacteriostatic activity, promoting dormancy and, thus, stress tolerance. However, in the current state of the literature, we think there is no strong evidence of TA-mediated stress responses. Previous research led in our group showed that deleting five TA systems did not lead to viability or fitness changes under various stress conditions, supporting the idea that these modules are not implicated in the stress response (28). Moreover, proteomic analysis of strains deleted for 10 TA systems failed to show changes in any protein levels, questioning the functionality of these systems beyond their own regulation and sustainability (36).

To conclude, our data do not support any implications of *mqsRA* and other toxin-antitoxin systems in core biological functions of E. coli, such as stress response and biofilm formation. We think that genetic modules presenting so much diversity and abundance and that raise so many questions deserve to be studied with due standards.

## MATERIALS AND METHODS

### Bacterial strains, plasmids, and growth conditions.

The constructs used in this study are detailed in Table S1. The oligonucleotides used in this study are detailed in Table S2. Details about these constructs are shown in Text S1. Briefly, fluorescent reporters were constructed by cloning promoters upstream of the *mScarlet-I* coding sequence (CDS) in pNF02, a single-copy pBeloBAC11 derivative. Complementation and overexpression vectors were based on pUA66, a low-copy-number vector.

10.1128/mBio.02678-19.5 TABLE S1 Strains used in this study. Download Table S1, DOCX file, 0.1 MB. Copyright © 2019 Fraikin et al. 2019 Fraikin et al. This content is distributed under the terms of the Creative Commons Attribution 4.0 International license.

10.1128/mBio.02678-19.6 TABLE S2 Oligonucleotides used in this study. Download Table S2, DOCX file, 0.1 MB. Copyright © 2019 Fraikin et al. 2019 Fraikin et al. This content is distributed under the terms of the Creative Commons Attribution 4.0 International license.

Unless specified otherwise, experiments were performed by inoculating a single colony in morpholinepropanesulfonic acid (MOPS) medium (76) supplemented with 0.4% glucose and grown overnight at 37°C with appropriate antibiotics (25 μg/ml kanamycin sulfate for *mqsA* overexpression strains and 15 μg/ml chloramphenicol for strains carrying fluorescent reporters). These cultures were diluted to an optical density at 600 nm (OD_600_) of 0.05 in fresh MOPS-glucose medium without antibiotics and grown to exponential phase (OD_600_, 0.4). Experiments using *mqsA* overexpression were performed in the same medium supplemented with 0.5 mM isopropyl-β-d-thiogalactoside (IPTG). Cells were treated and/or processed at this point. The lysogeny broth (LB) used at any point follows Lennox’s formulation (5 g/liter yeast extract, 10 g/liter tryptone, 5 g/liter NaCl).

### Flow cytometry analysis.

Cells grown to exponential phase were diluted to an OD_600_ of 0.01 and processed using an Attune NxT flow cytometer (Invitrogen) at a flow rate of 12.5 μl/min. When treated with deoxycholate, cells were first thoroughly washed with phosphate-buffered saline. Bacteria were separated from background noise by gating according to their forward- and side-scattering properties. Cell doublets were filtered out according to the pulse geometry (height versus area) of their side-scattering signal. The pulse height of mScarlet-I fluorescence was collected using a 561-nm solid-state laser and a 603/48-nm filter. Data were gathered, gated, and exported using the Attune NxT software 2.7.0, and FlowJo V10 was used to extract the geometric mean of the population for each experiment.

### MqsA stability analysis.

Cells were grown to OD_600_ of 0.4 in MOPS-glucose medium containing 0.5 mM IPTG and treated with stresses indicated in Fig. 2B. Rifampin (200 μg/ml) was also added at time zero to inhibit transcription and *de novo* MqsA synthesis. A volume of 9 ml of culture was sampled at given time points after treatment and mixed with 1 ml of ice-cold 50% trichloroacetic acid. After at least 30 min of incubation on ice, samples were pelleted, washed twice with 80% acetone, dried, resuspended in 200 μl of loading buffer (62.5 mM Tris-PO_4_ [pH 7.5], 3% sodium lauryl sulfate, 1 mM EDTA, 30% glycerol, 100 mM dithiothreitol, and 0.01% phenol red), and heated at 70°C for 20 min. Forty micrograms of protein was loaded on a Tris-Tricine gel, transferred to a polyvinylidene fluoride membrane, and then probed with a custom-made rabbit anti-MqsA antibody (Delphi Genetics) and a horseradish peroxidase (HRP)-conjugated anti-rabbit antibody (Calbiochem). MqsA was detected using enhanced chemiluminescence (SuperSignal West Femto; Thermo Fisher Scientific), and images were taken in an Odyssey Fc imager (Li-Cor Biosciences).

### MIC determination and killing assays.

MICs were determined by spotting 1 μl of 100×-diluted exponentially growing cultures on M9 gluconate plates (8.5 g/liter Na_2_HPO_4_·2H_2_O, 3 g/liter KH_2_PO_4_, 0.5 g/liter NaCl, 1 g/liter NH_4_Cl, 2 mM MgSO_4_, 0.4% gluconate, 11 g/liter agarose) containing linear increases of hydrogen peroxide (50 μM steps) or sodium deoxycholate (0.5% steps). MIC values were determined as the minimal concentration where no growth was visible after 24 h. Killing assays were performed by growing cells to an OD_600_ of 0.4 in MOPS-glucose medium before treating them with 20 mM hydrogen peroxide or 20% sodium deoxycholate and plating on LB plates at given time points. The viability at each time point was calculated as the number of colonies at a time point divided by the number of colonies before treatment.

### Biofilm microtiter plate assay.

Overnight cultures were diluted to an OD_600_ of 0.05 in LB medium, and 100-μl aliquots were dispensed in a flat-bottom polystyrene 96-well microtiter plate (catalog no. 655180; Greiner Bio-One). Plates containing six technical replicates of each strain tested were incubated statically at 28°C for 48 h. Nonadherent cells were gently washed out of the plate with distilled water. Adherent cells were then stained with 150 μl crystal violet (0.1%) for 15 min, thoroughly rinsed with distilled water, and submerged in 200 μl ethanol-acetone (4:1) for 15 min, after which adherent cells were suspended by pipetting. The quantity of adherent biomass was determined by measuring the absorbance of fixed crystal violet (*A*_595_) using a SpectraMAX i3 platform operated by SoftMAX Pro 3 (Molecular Devices).

### Congo red assays.

Congo red binding of planktonic cells was measured by growing cells overnight in LB medium, harvesting a quantity equivalent to 1 ml of overnight culture at an OD_600_ of 5, washing these cells with 1 ml tryptone water (1% tryptone), and then resuspending them in 1 ml of tryptone water with 40 μg/ml Congo red (and 0.5 mM IPTG when overexpressing *mqsA*). After 3 h of incubation at 28°C, cells were pelleted, and unbound Congo red was measured spectrophotometrically at 490 nm (*A*_490_). Bound Congo red fractions were calculated as the *A*_490_ of unbound Congo red subtracted from the *A*_490_ of tryptone water with 40 μg/ml Congo red. Absolute concentrations of Congo red were calculated using a standard curve of Congo red in tryptone water. All measurements were normalized to cell densities using OD_600_ measurements before pelleting the cells. Macrocolonies were obtained by spotting overnight cultures grown in LB medium on Congo red agar (0.5% yeast extract, 1% tryptone, 40 μg/ml Congo red, 20 μg/ml Coomassie blue G-250) and incubating the plates for 7 days at 28°C.

10.1128/mBio.02678-19.7 TEXT S1 Supplementary Materials and Methods. Download Text S1, DOCX file, 0.1 MB. Copyright © 2019 Fraikin et al. 2019 Fraikin et al. This content is distributed under the terms of the Creative Commons Attribution 4.0 International license.
