# Supplementary material for: Reassessing the Role of the Type II MqsRA Toxin-Antitoxin System in Stress Response and Biofilm Formation: mqsA Is Transcriptionally Uncoupled from mqsR
Source: mBio. 2019 Dec 17;10(6):e02678-19. doi: 10.1128/mBio.02678-19 (PMC6918082; doi:10.1128/mBio.02678-19)
Supplement: TABLE S1 [file mBio.02678-19-st001.docx]

**Table S1** : Strains used in this study

| **Strain** | **Genotype** | **Reference** |
| --- | --- | --- |
| MG1655 | *E. coli* K-12 F- λ- *ilvG rfb-50 rph-1* | (4) |
| BL21(DE3) | *E. coli* B *lon ompT gal* (λ DE3) *dcm hsdS* | Novagen |
| Δ*mqsRA* | MG1655 Δ*mqsRA*::*FRT* | This study |
| Δ10_LVM_ | MG1655 Δ*chpB*::*FRT* Δ*mazEF*::*FRT* Δ*relBE*::*FRT* Δ*yefM-yoeB*::*FRT* Δ*dinJ-yafQ*::*FRT* Δ*yafNO*::*FRT* Δ*prlF-yhaV*::*FRT* Δ*hicAB*::*FRT* Δ*higBA*::*FRT* Δ*mqsRA*::*FRT* | (2) |
| Δ10_AH_ | MG1655 Δ*hicAB*::*FRT* Δ*mqsR*::*FRT* Δ*yafO*::*FRT* Δ*yhaV*::*FRT* Δ*higB*::*FRT* Δ*yefM-yoeB* Δ*dinJ-yafQ* Δ*relBE* Δ*chpBS* Δ*mazF* | (5) |
| Δ*rpoS* | MG1655 Δ*rpoS*::*aphA2* | Lab collect. |
| Δ*tolC* | MG1655 Δ*tolC*::*FRT* | Lab collect. |
| Δ*csgA* | MG1655 Δ*csgA*::*aadA* | Lab collect. |
| **Plasmid** | **Properties** | **Reference** |
| pNF02 | oriF *cat* λt1ter-proDp-*mScarlet-I*-T7TE*luxI*A | (2) |
| p*RA* | pNF02 ΔproDp::p*mqsRA* | This study |
| p*A1* | pNF02 ΔproDp::p*mqsA1* | This study |
| p*A1+2* | pNF02 ΔproDp::p*mqsA1+2* | This study |
| p*csgD* | pNF02 ΔproDp::p*csgD* | This study |
| p*cspD* | pNF02 ΔproDp::p*cspD* | This study |
| p*rpoS* | pNF02 ΔproDp::p*rpoS* | This study |
| pUA66 | ori101 *aphA2* *gfp*-*rrnB*T1 | (6) |
| pUA-*mqsRA* | pUA66 Δ*gfp*::p*mqsRA*-*mqsRA* | This study |
| pLLU101 | pUA66 Δ*gfp*::*lacI*-p*lac*L8.UV5 | This study |
| pLLU-*mqsA* | pLLU101 *mqsA* | This study |
