# Supplementary material for: Reassessing the Role of the Type II MqsRA Toxin-Antitoxin System in Stress Response and Biofilm Formation: mqsA Is Transcriptionally Uncoupled from mqsR
Source: mBio. 2019 Dec 17;10(6):e02678-19. doi: 10.1128/mBio.02678-19 (PMC6918082; doi:10.1128/mBio.02678-19)
Supplement: TABLE S2 [file mBio.02678-19-st002.docx]

**Table S2** : Oligonucleotides used in this study.

| **Name** | **Sequence (5’-3’)** |
| --- | --- |
| *AvrII*-p*mqsRA* F | CCCGCCTAGGTGACTCCAGCTTCCCTTATAAATTCC |
| *NsiI*-p*mqsRA* R | CCCCATGCATAACCCCCGCCTCC |
| *NheI*-p*mqsA1* F | CCCCGCTAGCAAAAGCATGACCACCTACTCTGATCATACTATCTGG |
| *NheI*-p*mqsA1-2* F | ACCCGCTAGCAACGCACACCACATACACGTTTGAGTC |
| *NsiI*-p*mqsA* R | CCCCATGCATATTACTTCTCCTTAAACGAGACGATCAGTACGTCATG |
| *MluI*-p*rpoS* F | CCCCACGCGTTCCGTGACCTTGCTCAGCGC |
| *BmtI*-RBS-p*rpoS* R | CCCCGCTAGCTTTCTCCTCTTTCTCTAGTAGGCCTTTTCGTCAAAAACCTCAACTCCG |
| *XbaI*-p*csgD* F | CCCCTCTAGATTCTTCTTGCCCGTCGCTGATTGC |
| *BmtI*-RBS-p*csgD* R | CCCCGCTAGCTTTCTCCTCTTTCTCTGCACACCTGACAGCTGCCTC |
| *AvrII*-p*cspD* F | CCCACCTAGGCAAGAGCTTATTTGCCTGACCAATCTACTCC |
| *BmtI*-RBS-p*cspD* R | CCCCGCTAGCTTTCTCCTCTTTCTCTTTACCACTTAACAGTACCCTTTTCCATGCTTCG |
| *BamHI*-*mqsRA* F | CCCCGGATCCTGATGCCTGACTCCAGCTTCCC |
| *NheI*-*mqsRA* R | CCCCGCTAGCGAGTGATTTGGCTCACACTCCGG |
| *SalI*-*lacI* F | CCCCGTCGACCAGATCCCGGACACCATCG |
| *SpeI*-RBS-UV5 R | CCCCACTAGTGCACCCGGGAATTCTAGAAGCTTGTACATGCATGCTAGCTGTTTCCTGT  GTGAAATTGTTATCC |
| *NheI*-*mqsA* F | CCCCGCTAGCATGAAATGTCCGGTTTGCCACCAGG |
| *HindIII*-*mqsA* R | CCCCAAGCTTAACGGATTTCATTCAATAGTTCTGGATGCTTATCCAG |
