# Supplementary material for: Reassessing the Role of the Type II MqsRA Toxin-Antitoxin System in Stress Response and Biofilm Formation: mqsA Is Transcriptionally Uncoupled from mqsR
Source: mBio. 2019 Dec 17;10(6):e02678-19. doi: 10.1128/mBio.02678-19 (PMC6918082; doi:10.1128/mBio.02678-19)
Supplement: TEXT S1 [file mBio.02678-19-s0001.docx]

**Supplemental Materials and Methods**

**Plasmid and strain construction**

All vectors were constructed using standard restriction-ligation protocols. Polymerase chain reactions were performed using Q5 DNA polymerase (New England Biolabs).

Fluorescent reporters are based on pNF02, a single copy plasmid constructed by inserting synthetic fragments in pBeloBAC11. This vector encodes the *mScarlet-I* fluorescent reporter under the control of the strong proDp synthetic promoter (1), all of which is flanked with terminators. Promoters are cloned upstream of the *mScarlet-I*-encoding gene using restriction enzymes *MluI* or *AvrII* in 5’ and *BmtI* or *NsiI* in 3’. The p*RA* plasmid was constructed by amplifying the *mqsRA* promoter from *E. coli* MG1655 using primers *AvrII*-p*mqsRA* F and *NsiI*-p*mqsRA* R (Table S2). This amplification fragment was digested with *AvrII* and *NsiI* and ligated in pNF02 digested with the same enzymes. Similarly, p*A1* and p*A1+2* plasmids were constructed by amplifying respective promoters with primers *NheI*-p*mqsA1* F, *NheI*-p*mqsA1-2* F and *NsiI*-p*mqsA* R (Table S2), amplification fragments were digested with *NheI* and *NsiI* and ligated in pNF02 digested with *AvrII* and *NsiI*. The resulting reporter plasmids contain the aforementioned promoters up to the start codon of *mqsR* (p*RA*) or *mqsA* (p*A1*, p*A1+2*). The p*csgD* reporter plasmid was constructed by amplifying the *csgD* promoter from *E. coli* MG1655 using primers *XbaI*-p*csgD* F and *BmtI*-RBS-p*csgD* R (Table S2). This fragment was restricted with *XbaI* and *BmtI* and ligated in pNF02 digested with *AvrII* and *BmtI*. Similarly, p*cspD* and p*rpoS* plasmids were constructed by amplifying respective promoters with primers *AvrII*-p*cspD* F, *BmtI*-RBS-p*cspD* R, *MluI*-p*rpoS* F and *BmtI*-p*rpoS* R (Table S2), restriction with *ad-hoc* enzymes and ligation in pNF02 digested with *AvrII* and *BmtI*. These three vectors contain a synthetic ribosome binding site before the start codon of *mScarlet-I* (AGAGAAAGAGGAGAAAGCTAGCATG).

Complementation and overexpression vectors were based on pUA66, a well-insulated and compact plasmid encoding the *gfp* gene. To construct pUA-*mqsA*, we replaced *gfp* with the *mqsRA* operon (all promoters included) amplified using primers *BamHI-mqsRA* F and *NheI-mqsRA* R (Table S2). This fragment was digested with *BamHI* and *NheI* then ligated in pUA66 restricted with *BamHI* and *XbaI*. pLLU101 was constructed by amplifying a *lacI*-p*lac*L8.UV5 fragment from the DE3 prophage encoded in *E. coli* BL21(DE3) with primers *SalI*-*lacI* F and *SpeI*-RBS-UV5 (Table S2). This fragment was digested with *SalI* and *SpeI* then ligated in pUA66 restricted with *XhoI* and *XbaI*. pLLU101 contains a multiple cloning site in 3’ of p*lac*L8.UV5 (*BmtI*-*NsiI*-*BsrGI*-*HindIII*-*XbaI*-*EcoRI*-*XmaI*). pLLU-*mqsA* was constructed by amplifying *mqsA* from *E. coli* MG1655 using primers *NheI*-*mqsA* F and *HindIII*-*mqsA* R (Table S2). This fragment was digested with *NheI* and *HindIII* then ligated in pLLU101 restricted with the same enzymes. The Δ*mqsRA* strain was constructed as described for the Δ10_LVM_ in a previous study (2).

**Catalase activity assay**

Catalase activity measurements were performed by treating exponentially growing cells with 60 mM hydrogen peroxide and titrating consumed peroxide with acidic dichromate (75 % acetic acid, 1.25 % potassium dichromate) after 5, 10 and 15 min to form Cr^3+^ (3). Under boiling, Cr3+ can react with acetic acid to yield chromic acetate, which can be quantified by spectrophotometry at 570 nm. After linear regression of sampled timepoints and establishment of a standard hydrogen peroxide curve, catalase activity was expressed as the number of consumed micromoles of hydrogen peroxide per minute (one unit), per milliliter of culture normalized culture turbidity (OD_600_).

**Supplementary References**

1. Davis JH, Rubin AJ, Sauer RT. 2010. Design, construction and characterization of a set of insulated bacterial promoters. Nucleic Acids Research 39:1131–1141.

2. Goormaghtigh F, Fraikin N, Putrinš M, Hallaert T, Hauryliuk V, Garcia-Pino A, Sjödin A, Kasvandik S, Udekwu K, Tenson T, Kaldalu N, Van Melderen L. 2018. Reassessing the Role of Type II Toxin-Antitoxin Systems in Formation of Escherichia coli Type II Persister Cells. mBio 9:e00640-18.

3. Sinha AK. 1972. Colorimetric assay of catalase. Analytical Biochemistry 47:389–394.

4. Blattner FR, Plunkett G, Bloch CA, Perna NT, Burland V, Riley M, Collado-Vides J, Glasner JD, Rode CK, Mayhew GF, Gregor J, Davis NW, Kirkpatrick HA, Goeden MA, Rose DJ, Mau B, Shao Y. 1997. The Complete Genome Sequence of Escherichia coli K-12. Science 277:1453.

5. Harms A, Fino C, Sørensen MA, Semsey S, Gerdes K. 2017. Prophages and Growth Dynamics Confound Experimental Results with Antibiotic-Tolerant Persister Cells. mBio 8:e01964-17.

6. Zaslaver A, Bren A, Ronen M, Itzkovitz S, Kikoin I, Shavit S, Liebermeister W, Surette MG, Alon U. 2006. A comprehensive library of fluorescent transcriptional reporters for Escherichia coli. Nature Methods 3:623–628.

7. Christensen-Dalsgaard M, Jørgensen MG, Gerdes K. 2010. Three new RelE-homologous mRNA interferases of Escherichia coli differentially induced by environmental stresses. Molecular Microbiology 75:333–348.

8. Wang X, Kim Y, Hong SH, Ma Q, Brown BL, Pu M, Tarone AM, Benedik MJ, Peti W, Page R, Wood TK. 2011. Antitoxin MqsA helps mediate the bacterial general stress response. Nature Chemical Biology 7:359.

9. Jubelin G, Vianney A, Beloin C, Ghigo J-M, Lazzaroni J-C, Lejeune P, Dorel C. 2005. CpxR/OmpR Interplay Regulates Curli Gene Expression in Response to Osmolarity in Escherichia coli. J Bacteriol 187:2038.

10. Yamanaka K, Inouye M. 1997. Growth-phase-dependent expression of cspD, encoding a member of the CspA family in Escherichia coli. J Bacteriol 179:5126.

11. Mika F, Hengge R. 2005. A two-component phosphotransfer network involving ArcB, ArcA, and RssB coordinates synthesis and proteolysis of σS (RpoS) in E. coli. Genes & Development 19:2770–2781.
